# Abnormal ambiguous facial expression recognition in Chinese patients with schizophrenia

**DOI:** 10.1186/s12888-024-05685-4

**Published:** 2024-03-26

**Authors:** Xiaoli Lyu, Yuyan Chi, Zhenyu Wang, Xinyan Shao, Guangya Zhang, Chuanwei Li, Chenglong Dong, Xuqin Wang, Xin Li, Chuanlin Zhu, Xiaofeng Xu, Xiangdong Du

**Affiliations:** 1 Affiliated WuTaiShan Hospital of Medical College of Yangzhou University, 225003 Yangzhou, Jiangsu China; 2 Suzhou Guangji Hospital, The Affiliated Guangji Hospital of Soochow University, 215137 Suzhou, Jiangsu China; 3 Wujiang District Mental Rehabilitation Hospital, 215200 Suzhou, Jiangsu China; 4 School of Educational Science, Yangzhou University, 225002 Yangzhou, Jiangsu China

**Keywords:** Ambiguous facial expression recognition, Schizophrenia, Background information

## Abstract

**Background:**

Patients with schizophrenia (SCZ) exhibit difficulties deficits in recognizing facial expressions with unambiguous valence. However, only a limited number of studies have examined how these patients fare in interpreting facial expressions with ambiguous valence (for example, surprise). Thus, we aimed to explore the influence of emotional background information on the recognition of ambiguous facial expressions in SCZ.

**Methods:**

A 3 (emotion: negative, neutral, and positive) × 2 (group: healthy controls and SCZ) experimental design was adopted in the present study. The experimental materials consisted of 36 images of negative emotions, 36 images of neutral emotions, 36 images of positive emotions, and 36 images of surprised facial expressions. In each trial, a briefly presented surprised face was preceded by an affective image. Participants (36 SCZ and 36 healthy controls (HC)) were required to rate their emotional experience induced by the surprised facial expressions. Participants’ emotional experience was measured using the 9-point rating scale. The experimental data have been analyzed by conducting analyses of variances (ANOVAs) and correlation analysis.

**Results:**

First, the SCZ group reported a more positive emotional experience under the positive cued condition compared to the negative cued condition. Meanwhile, the HC group reported the strongest positive emotional experience in the positive cued condition, a moderate experience in the neutral cued condition, and the weakest in the negative cue condition. Second, the SCZ (vs. HC) group showed longer reaction times (RTs) for recognizing surprised facial expressions. The severity of schizophrenia symptoms in the SCZ group was negatively correlated with their rating scores for emotional experience under neutral and positive cued condition.

**Conclusions:**

Recognition of surprised facial expressions was influenced by background information in both SCZ and HC, and the negative symptoms in SCZ. The present study indicates that the role of background information should be fully considered when examining the ability of SCZ to recognize ambiguous facial expressions.

## Introduction

Facial expression recognition is one of the most basic skills of social cognition, serving as the primary means to understand the emotional states of others in daily human interaction [1, 2]. Facial expressions represent a complex stimuli that conveys a variety of important messages in social interaction. Deficits and biases in facial expression recognition are linked to impairments in social and emotional functions, which exacerbate mental disorders and negatively impact treatment outcomes [3–6].

Previous researchers have conducted studies on facial expression recognition in many psychiatric disorders, particularly in SCZ. SCZ showed impaired facial expression recognition, especially for negative emotions such as sadness, fear, and disgust, which were more impaired compared to positive emotions [7, 8]. It is difficult for SCZ to judge the real emotional state of others, making their social interactions more challenging. This kind of impairment has been reported in the early stages of the disease and affects the recognition of various emotional states [9, 10]. This can seriously interfere with the social functioning of individuals and cause inappropriate social reactions [11]. Bulgari et al. [12] found that impairment in facial expression recognition among SCZ was one of the causes of aggressive behavior. Therefore, communication between SCZ and the general society is inseparable from the process of facial expression recognition [13]. The ability to deal with facial expressions is not only a core component of their social cognition but also an important factor affecting social function.

Some studies have shown that abnormal processing of facial expressions in SCZ was related to clinical symptoms [14–18], but the research results were not consistent. Mandal et al. [17] compared the facial expressions (happiness, sadness, and neutral) recognition characteristic of SCZ with those of HC, and found that SCZ along with negative symptoms exhibited a generalized emotion recognition deficit. SCZ who exhibited positive symptoms showed a deficit in their recognition of sad emotion. However, Baudouin et al. [19] reported that disorders of facial expression processing in SCZ were associated with negative symptoms but not with positive symptoms. It has also been reported that the facial expression recognition abnormality in SCZ exists stably throughout the illness and does not change significantly due to differences in illness period, clinical symptoms and severity [8]. Meanwhile, Tseng et al. [20] found that the accuracy of recognizing happy facial expressions in SCZ was negatively correlated with the PANSS total score, delusion/hallucination, and hostility/excitement factors. Additionally, previous studies have shown that when participants were required to complete a facial expression recognition task (in a single trial, where only facial expression was present and no context was provided), the response speed of the SCZ group was slower than that of HC group [16, 21, 22]. Overall, compared with healthy participants, SCZ showed deficits in facial expression recognition.

In the studies mentioned above, the valence of the target facial expressions was unambiguous. Valence refers to a pleasure/unpleasure emotional experience and its strength, and its score ranges from 1 (very unpleasant) to 9 (very pleasant). Facial expressions categorized by valence include positive, negative, and neutral. However, there are also some emotions with uncertain valence (for example, surprise), and the emotional experience brought by surprise is closely related to the situation [23–25]. From a psychological point of view, surprise is often associated with the difference between an individual’s expectations and reality. For example, someone may be surprised to be told that she/he have received a large bonus; or she/he may be surprised to be told that one of her/his good friends died suddenly, considering she/he was so young. Although individuals may be surprised in both situations, the nature of their surprise clearly differs; happiness is more likely in the former, while shock is more likely in the latter. It can thus be seen how background information may affect the processing of emotions, but researchers often ignored this in previous studies.

From the perspective of the emotional dimension, the valence categories of some emotions are easy to judge. For example, individuals can generally classify “happiness” and “sadness” as positive and negative emotions, respectively. However, when individuals judge the valence attribute of surprise, it is difficult to obtain a consistent answer. Surprise is an emotion with a duration shorter than one second [2]. Depending on the trigger, this emotion can easily transition to a different emotion, Therefore, it can be said that surprise is actually a transitional emotion [26, 27]. A large number of studies have shown that the valence of surprised facial expressions is uncertain and it is affected by context [23, 25, 28–30]. Thus, surprise is a less accurately identified emotion than other basic emotions (such as happiness and fear).

A large number of studies have investigated the performance of SCZ in processing facial expression with unambiguous emotional valence, achieving significant results. However, a review showed a research gap in China regarding the influence of background information on the recognition of surprised facial expressions in this population. For SCZ, communication with society cannot be separated from the process of facial emotion recognition. Therefore, studying the influence of background information on the recognition of ambiguous emotions by SCZ may be more helpful for their social function recovery, and is also expected to provide valuable insights for clinical rehabilitation treatment. The present study aimed to investigate the influence of emotional background information on the recognition of ambiguous facial expressions in SCZ. A 3 (emotion: negative, neutral, and positive) × 2 (group: SCZ and HC) experimental design was adopted. Participants were required to complete the emotional experience rating task (please refer to the Procedure section for more details). Three sub-goals were included. (1) The first sub-goal was to explore whether participants’ performance in completing the emotional experience rating task would be influenced by background information. More specifically, it aimed to investigate whether emotional experience rating scores differed significantly under negative, neutral, and positive background conditions. (2) The second sub-goal was to explore whether there are differences in response speed between SCZ and HC when completing the emotional experience rating task. (3) The third sub-goal was to explore the relationship between the performance of SCZ in processing surprised facial expressions and their clinical symptoms.

## Methods

### Participants

A priori power analysis conducted by G*Power 3.1.9 [31] showed that a total sample size of 28 participants was needed to achieve a satisfactory effect size (α = 0.05, effect size f = 0.25, power level = 0.95). To ensure that our sample size was sufficiently large, thirty-six HC and 36 SCZ were recruited.

Details of the participants were as follows. Firstly, thirty-six schizophrenia inpatients (17 females, M ± SD, 40.22 ± 11.59 years old) were recruited from Suzhou Guangji Hospital. The inclusion criteria were: (1) a diagnosis of schizophrenia according to the Diagnostic and Statistical Manual of Mental Disorders, fifth edition. (2) aged between 18 and 60 years old. (3) all inpatients must have been receiving stable antipsychotic treatment at the time of the study. (4) no history of drug and other substance abuse. (5) female subjects must not be breastfeeding or pregnant. (6) no other physical illness affecting cognitive function. (7) right-handed. Psychopathological assessment was based on the Positive and Negative Syndrome Scale (PANSS) [32]. Secondly, thirty-six healthy right-handed controls (20 females, M ± SD, 19.22 ± 0.59 years old) were recruited from Yangzhou University. All participants had normal or corrected-to-normal visual acuity and no history of brain trauma or mental illness. None of them had participated in similar studies before. All participants were paid for their participation. All participants signed an informed consent form before completing the experimental task, which was in accordance with the Declaration of Helsinki (1991). The present study was approved by the Clinical Research Ethics Committee of Suzhou Guangji Hospital (approval number: 2022-007).

The average age of SCZ (M ± SD, 40.22 ± 11.59 years old) was older than that of HC (19.22 ± 0.59 years old), t = 10.857, df = 35, *p* < 0.001. The education level of SCZ (11.389 ± 3.236 years) was lower than that of HC (14.222 ± 0.591 years), t = 5.073, df = 35, *p* < 0.001. There was no statistical difference in gender between the two groups (χ^2^ = 0.500, *p* = 0.479). Most of the SCZ (80.56%) were medicated with atypical neuroleptics. Most medications were administered orally, with two SCZ treated with a combination of long-acting injectable antipsychotics. The total PANSS score for SCZ was 59.03 ± 8.23, with a positive symptom score of 10.56 ± 3.77, a negative symptom score of 21.83 ± 5.76 and a general psychopathology score of 26.64 ± 5.74.

### Materials

The experimental materials consisted of 108 affective pictures (36 negative, 36 neutral, and 36 positive) and 36 images of surprised facial expressions. The affective pictures used in this study were selected from the native Chinese Affective Picture System (CAPS) [33]. The numbers of affective pictures in the CAPS database were as follows: negative: 142, 146, 149, 150, 152, 158, 170, 179, 180, 181, 199, 203, 205, 219, 222, 246, 251, 268, 269, 502, 511, 526, 532, 539, 542, 544, 545, 549, 555, 559, 571, 572, 573, 577, 587, 597; neutral: 297, 298, 301, 316, 347, 367, 376, 378, 383, 398, 403, 407, 415, 416, 419, 426, 477, 649, 665, 674, 682, 698, 705, 706, 709, 711, 725, 732, 733, 735, 741, 742, 743, 744, 745, 746; positive: 7, 11, 12, 13, 14, 18, 20, 72, 73, 77, 85, 101, 139, 430, 448, 453, 467, 468, 478, 488, 489, 640, 652, 656, 660, 663, 675, 684, 685, 691, 701, 718, 776, 780, 781, 814. Separate one-way ANOVAs were conducted for valence and arousal. The main effect of valence was significant, F(2,107) = 2309.56, *p* < 0.001. The valence of positive pictures (M ± SD, 7.34 ± 0.33) was higher (*ps* < 0.001) than that of negative (2.41 ± 0.34) and neutral (5.45 ± 0.25) pictures, and the valence of neutral pictures was higher than that of negative ones (*p* < 0.001). The main effect of arousal was significant, F(2,107) = 160.43, *p* < 0.001. The arousal of positive pictures (M ± SD, 5.65 ± 0.50) and negative pictures (5.83 ± 0.74) was higher than neutral pictures (3.71 ± 0.37), while there was no significant difference between positive and negative pictures.

The facial expression images were selected from the NimStim Database [34]. Referring to previous studies [35–37], considering the influence of cultural differences, to ensure that most participants could recognize the facial expressions, the original images were rated before the formal experiment. Before the formal experiment, thirty participants, who did not take part in the formal experiment, were required to complete the facial expression-labeling task. The order in which the stimuli were presented in each trial was as follows: a white “+” (500 ms), a facial expression image (1000 ms), the facial expression-labeling task (unlimited), the certainty rating task (unlimited), followed by a blank (200 ms). In the facial expression-labeling task, participants were required to select which emotion the facial expression image was showing from seven alternative answers (anger, fear, sadness, disgust, surprise, neutral, or happiness), by pressing the corresponding key on the keyboard. In the certainty rating task, participants were required to report their certainty level when completing the facial expression-labeling task, by using a 9-point scale (“1” stands for very uncertain, “9” stands for very certain). Finally, the average recognition rate of each image and the corresponding certainty level were calculated. Only images with a mean recognition rate higher than 85%, and a median of certainty level higher than 8 were adopted for the formal experiment. The number of the adopted models in the NimStim database was as follows: female, 1, 2, 3, 5, 6, 7, 8, 9, 10, 11, 12, 13, 14, 15, 16, 17, 18, 19; male, 21, 22, 23, 24, 25, 26, 27, 28, 29, 30, 32, 33, 34,35, 36, 37, 41, 42.

### Procedure

**Fig. 1:**
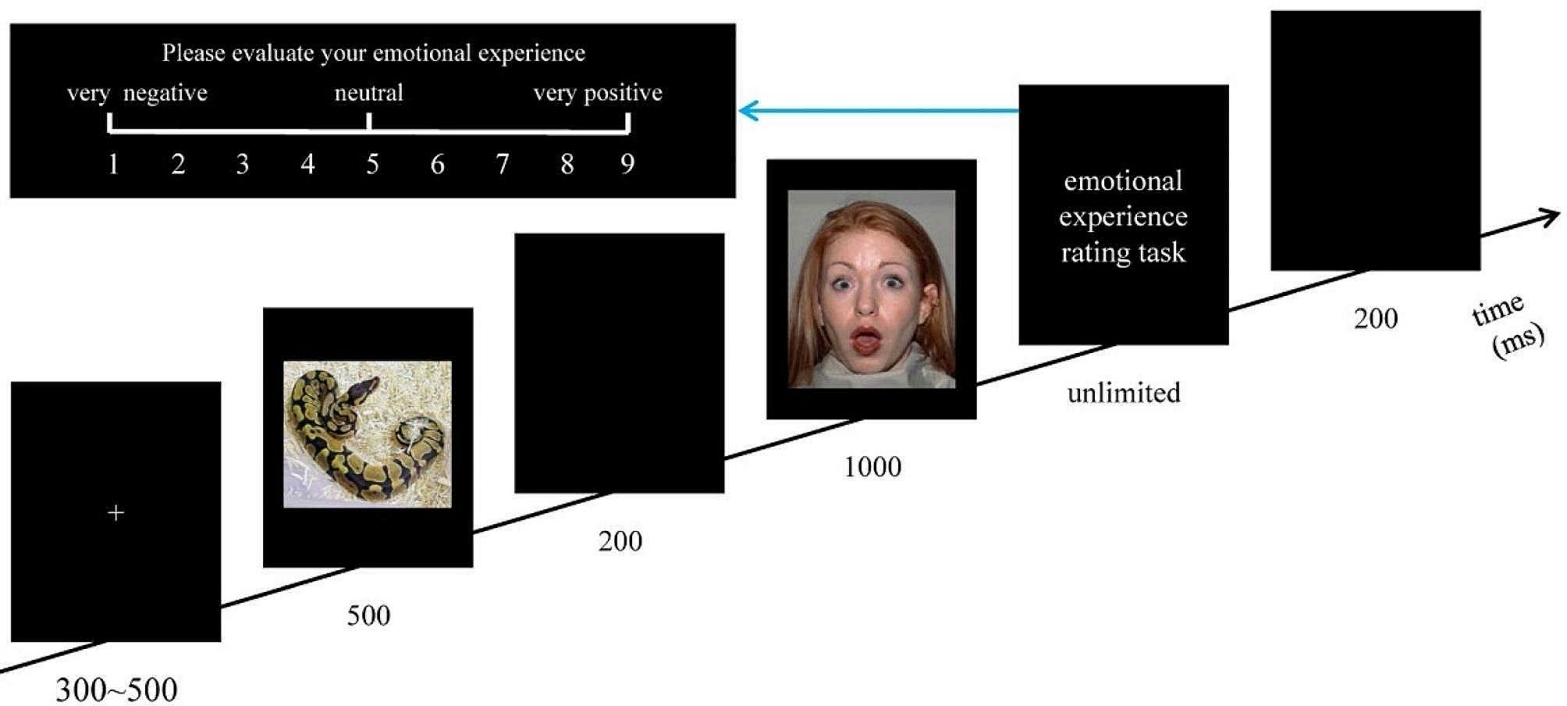
The sequence of one trial

The procedure of this study was programmed with E-prime 2.0 (Psychology Software Tools Inc., Pittsburgh, PA, USA). As shown in Fig. 1, the order in which the stimuli were presented in each trial was as follows: each trial started with a white“+” (300 ms ∼ 500 ms), followed by the cue stimulus (an affective picture, 500 ms), then a blank (200ms), the target stimulus (a surprised facial expression image, 1000 ms), the emotional experience rating task (unlimited), and finally another blank (200 ms). All of the stimuli were presented in the center of the screen, and the background color of the screen was black. In the emotional experience rating task, participants were required to report their emotional experience elicited by the target stimulus, using a 9-point rating scale. “1” representing “very negative”, “5” representing “neutral”, and “9” representing “very positive”. All participants were told to respond as accurately and quickly as possible. A pseudo-random design was adopted in this study, and each experimental condition would not appear more than 3 times in succession.

To ensure the participants successfully understood the experimental process, twelve practice trials were provided before completing the formal experiment for each participant. The experimental materials in these practice trials were not used in the formal experiment. In order to reduce the effect of fatigue, all participants were required to rest for two minutes after each block.

### 2.4 Data analysis

The experimental data analysis was conducted using SPSS 26.0. The results of emotional experience rating and participants’ reaction time (RT) have been analyzed by adopting analysis of variances (ANOVAs). Dependent variables included participants’ emotional experience rating scores and RTs for completing the emotional experience rating task; independent variables comprised the cue stimuli types (negative, neutral, and positive) and group (HC and SCZ). The cue stimulus type was a within-subject variable, while group was a between-subject variable. The Greenhouse-Geisser correction was applied to the *p* values when the degree of freedom did not satisfy the spherical test hypothesis. All post-hoc tests included Bonferroni’s correction. Partial eta-squared (η2 p) was used to describe effect sizes.

## Results

### The emotional experience rating results

The results of 3 (cue type: negative, neutral, and positive) × 2 (group: HC and SCZ) ANOVA showed that the main effect of cue type was significant, F (2, 216) = 52.061, *p* < 0.001, η2 *p* = 0.331. The post-hoc analysis showed that the emotional experience rating scores under positive cue condition (M ± SD, 5.97 ± 1.10) were higher (*ps* < 0.001) than those under neutral (5.23 ± 0.78) and negative (4.23 ± 1.12) ones, while the corresponding scores under neutral cue condition were higher (*p* < 0.001) than those under negative cue condition.

**Fig. 2:**
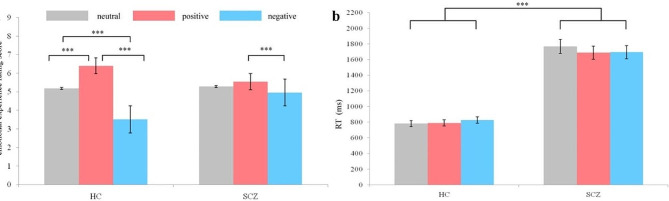
The emotional experience rating results (**a**) and RT of completing the emotional experience rating task (**b**). “***” means “*p* <.001”. Error bars represent standard errors. HC = healthy control, SCZ = patients with schizophrenia. Grey bar = neutral cue, red bar = positive cue, blue bar = negative cue

The interaction of cue type and group was significant, F (2, 216) = 22.960, *p* < 0.001, η2 *p* = 0.179. Simple effect analysis showed that the emotional experience rating scores under positive cue condition (6.40 ± 0.92) were higher (*ps* < 0.001) than those under neutral (5.18 ± 0.52) and negative (3.51 ± 1.13) ones, while the corresponding scores under neutral cue condition were higher (*p* < 0.001) than under negative condition for HC. Meanwhile, the emotional experience rating scores under positive cue condition (5.54 ± 128) were higher (*p* = 0.049) than those under negative (4.96 ± 1.11) ones for SCZ. There were no statistical differences in the emotional experience scores of SCZ when comparing either positive and neutral cues or neutral and negative cues (see Fig. 2a). The main effect of group was not significant, F (1, 216) = 2.750, *p* = 0.099, η2 *p* = 0.013.

### The RT of completing the emotional experience rating task

The results of 3 (cue type: negative, neutral, and positive) × 2 (group: HC and SCZ) ANOVA showed that the main effect of group was significant, F(1, 216) = 68.961, *p* < 0.001, η2 *p* = 0.247. The post-hoc analysis showed that the mean RT of the HC group (M ± SD, 800.182 ± 218.84) was shorter than that of the SCZ group (1717.529 ± 1124.21). The main effect of cue type was not significant, F(2, 216) = 0.036, *p* = 0.965, η2 *p* < 0.001. The interaction of cue type and group was not significant, F (2, 216) = 0.105, *p* = 0.900, η2 *p* = 0.001.The results were showed in Fig. 2b.

### 3.3 Correlation between schizophrenia symptoms and emotional experience scores

The results of the study showed that the severity of schizophrenia symptoms measured with PANSS (negative symptoms - N) was negatively associated with emotional experience scores under neutral and positive background information. No significant correlation between age and emotional experience scores was found in both groups (see Table 1).

**Table 1:** Correlation between schizophrenia symptoms and emotional experience scores

	neutral	positive	negative
PANSS total score	-0.034	-0.032	-0.113
P subscore	0.242	0.228	-0.031
N subscore	-0.382*	-0.304^*^	-0.195
G subscore	0.177	0.145	0.054
age			
SCZ	0.070	0.162	0.103
HC	0.004	0.044	-0.038

*p<0.05, PANSS = Positive and Negative Syndrome Scale, P = PANSS positive symptom subscale, N = PANSS negative symptom subscale, G = PANSS general psychopathology subscale. SCZ = patients with schizophrenia, HC = healthy control

## Discussion

To the best of our knowledge, this study is the first to examine the effect of emotional background information on the recognition of surprised facial expressions in SCZ in China. The results showed that SCZ can benefit from background information when asked to recognize surprised facial expressions. The emotional experience rating scores elicited by surprised facial expressions were similar between HC and SCZ when emotional background information was available.

Under the positively cued condition, a positive bias toward surprised facial expression was observed, whereas a negative bias was seen under the negatively cued condition. Previous studies on facial expression recognition in SCZ have predominantly focused on facial expressions with unambiguous valence, such as “anger” and “sadness”, these studies have confirmed that SCZ exhibit impairments in facial expression recognition [11, 12, 18]. In the absence of background information, SCZ showed impairment in recognizing surprised facial expressions, and the intensity of their emotional experience was lower than that of HCs [38]. Previous studies [39, 40] have explored the effects of verbal information and picture background information on the recognition of surprised facial expression in SCZ, indicating that their ability to process background information was intact. Our study was consistent with previous studies to some extent, however we found that SCZ showed abnormal processing of surprised facial expression under emotionally cued conditions.

Our study found that the emotional experience rating scores of surprised facial expressions in SCZ were higher in the positively cued condition than in the negatively cued condition. However, there was no difference in the emotional experience rating scores generated under the neutral cued condition compared with those under the positive and negative cued conditions. Lee et al. [40] found that in the absence of background information, both SCZ and HCs perceived fearful faces as showing more intense expressions of fear than neutral faces, and surprised faces as showing more intense expressions of surprise than neutral faces. They also rated faces expressing neutrality or surprise as conveying more fear when presented with fear-inducing sentences. Similarly, they rated these faces as conveying more surprise when presented with surprise-inducing sentences. Contextual information regulates the perception of surprised faces more strongly than that of neutral faces. Muros et al. [14] found that SCZ reported less intense emotional experience for negative and neutral emotions compared with positive emotions. Meanwhile, Kohler et al. [41] found that identification of several negative emotions, such as sadness and anger, was not impaired in SCZ. Additionally, Modinos et al. [42] investigated the neural correlates of emotional salience in HC, first-episode of psychosis (FEP) patients, and participants at ultra-high risk for psychosis (UHR), as well as its association with psychotic symptoms. Participants were required to rate their subjective emotional arousal in response to negative, neutral and positive pictures (selected from the International Affective Picture System). Meanwhile, both positive and negative symptoms were assessed for FEP patients and UHR. The results showed that compared with HC, the FEP and UHR groups exhibited increased subjective emotional arousal when processing neutral stimuli and reduced activation in the inferior frontal gyrus/anterior insula, dorsomedial prefrontal cortex, and amygdala when processing positive and negative stimuli. Additionally, corticolimbic hyperactivity in response to neutral stimuli was associated with higher levels of positive symptoms. These findings suggested that the difference in emotional experience ratings between SCZ group and HC in the present study may be more related to the SCZ group’s arousal in response to the cue, or aberrant emotional salience, which was supported by a recent study [43].

It is crucial to comprehensively investigate the emotional recognition characteristics of SCZ, as the conclusion drawn from expressions with unambiguous emotional valence [16, 21, 22] may not be sufficient to comprehensively describe the emotional recognition ability of SCZ, especially when facing ambiguous emotional information. The present study showed that SCZ responded slower than HC when they were required to complete the facial expression processing task. A previous study showed that when completing facial expressions recognition tasks with neutral, fear, anger, disgust, joy, and sadness target expressions, the RT of SCZ was longer than that of HC [14], which supported our results. Previous studies have shown that SCZ exhibited aberrant emotional salience [42]. For example, Pomarol-Clotet et al. mentioned that SCZ showed an impaired ability in judging the intensity of emotional stimuli [22]. However, Kohler et al. [41] found that participants’ performance in the facial expression recognition task was influenced by intensity. Specifically, participants (both SCZ and HC) performed better when recognizing high (vs. low) intensity expressions. Therefore, the longer RT observed in SCZ patients may be related to their arousal in response to the cue, or aberrant emotional salience.

Our study found that the more severe the negative symptoms in SCZ, the more impaired their emotional experience of facial expressions of surprise in the context of neutral and positive background information. Previous studies on the relationship between facial expression perception in SCZ and psychotic symptoms have been inconsistent. Additionally, the results of a meta-analysis by Chan et al. [44] showed that only negative symptoms (as measured by the PANSS) were found to be associated with facial expression perception in SCZ, and that those with severe negative symptoms showed more severe deficits in facial expression perception. Kitoko et al. [45] and Leszczynska et al. [46] also found a relationship between facial expression recognition deficits and the severity of negative symptoms in SCZ. Some studies found that facial expression recognition was associated with positive symptoms [2, 47], while other studies have not found any relationship between facial expression recognition and positive or negative symptoms [48].

Previous studies mainly focused on studying the ability of SCZ to recognize facial expressions with unambiguous valence (such as expressions of sadness and fear). The present study indicated that recognition of surprised facial expressions was influenced by background information in both SCZ and HC, as well as by the presence of negative symptoms in SCZ. Additionally, the response speeds of SCZ were slower than those of HC in recognizing facial expressions with ambiguous emotional valence. Such findings contribute to expanding our understanding of facial expression recognition performance in SCZ, which may serve as a reference for clinical diagnosis and guide the development of targeted treatment strategies, ultimately aiding in the improvement of their emotional recognition ability. Nevertheless, there were some limitations in the present study. This study was conducted during the coronavirus epidemic, due to the impact of the coronavirus, it was difficult to match the age and education level between the SCZ group and the HC group. Some studies [49, 50] have shown that emotional recognition can be affected by age, but there was a significant difference in age between SCZ and HC in this study. Therefore, the results of the present study should be interpreted cautiously. Additionally, due to practical constraints, the HCs recruited in this study only consisted of college students. Fortunately, previous study [51] have shown that the impairment in social background information processing among SCZ was not influenced by education level.

## Conclusion

In conclusion, the present study suggested that the recognition of ambiguous facial expressions in SCZ was influenced by background information. However, these patients were not able to distinguish between ambiguous expressions when influenced by positive versus neutral cues, or neutral versus negative cues. These findings may have practical implications for the treatment of social dysfunction in this population.

## Data Availability

The datasets used and/or analyzed during the current study are available from the corresponding author on reasonable request.

## Abbreviations

SCZ: Patients with schizophrenia
ANOVAs: Analysis of variances
HC: Healthy controls
RTs: Reaction times
